# Eye bank versus surgeon prepared DMEK tissues: influence on adhesion and re-bubbling rate

**DOI:** 10.1136/bjophthalmol-2020-317608

**Published:** 2020-10-26

**Authors:** Vito Romano, Ahmed Kazaili, Luca Pagano, Kunal Ajit Gadhvi, Mitchell Titley, Bernhard Steger, Luis Fernández-Vega-Cueto, Alvaro Meana, Jesus Merayo-Lloves, Ponzin Diego, Riaz Akhtar, Hannah J Levis, Stefano Ferrari, Stephen B Kaye, Mohit Parekh

**Affiliations:** 1 Instituto Universitario, Universidad de Oviedo and Fundacion de Investigacion Oftalmologica, Oviedo, Spain; 2 Ophthalmology, Royal Liverpool University Hospital, Liverpool, UK; 3 Eye and Vision Science, Institute of Life Course and Medical Sciences, University of Liverpool, Liverpool, UK; 4 Department of Biomedical Engineering, College of Engineering, University of Babylon, Hillah, Iraq; 5 Babylon Health Directorate, Ministry of Health, Babylon, Iraq; 6 Department of Mechanical, Materials and Aerospace Engineering, University of Liverpool School of Engineering, Liverpool, UK; 7 Department of Ophthalmology, Medical University of Innsbruck, Innsbruck, Austria; 8 International Centre for Ocular Physiopathology, Fondazione Banca Degli Occhi del Veneto, Venezia Zelarino, Italy; 9 UCL, London, UK

**Keywords:** Cornea, Treatment Surgery

## Abstract

**Aim:**

To investigate the difference in adhesion and rebubbling rate between eye bank and surgeon prepared Descemet membrane endothelial keratoplasty (DMEK) tissues.

**Methods:**

Laboratory and clinical retrospective comparative interventional case series. Research corneal tissues were obtained for laboratory investigation. The clinical study involved patients with endothelial dysfunction who underwent DMEK surgery and tamponade with air. Tissues were stripped using a standard DMEK stripping technique (SCUBA) and shipped as prestripped or loaded in a 2.2 intra-ocular lens cartridge with endothelium facing inwards (preloaded) before transporting from the eye bank to the surgeon. For surgeon prepared tissues, all the grafts were stripped in the theatre and transplanted or stripped in the laboratory and tested immediately. Adhesion force and elastic modulus were measured in the centre and mid-periphery in a laboratory ex vivo investigation using atomic force microscopy, while rebubbling rates were recorded in the clinical study.

**Results:**

There was no difference in endothelial cell viability between surgeon or eye bank prepared tissue. Surgeon-stripped DMEK grafts in the laboratory investigation showed significantly higher elastic modulus and adhesion force compared to prestripped and preloaded tissues (p<0.0001). In the clinical data, rebubbling rates of 48%, 40% and 15% were observed in preloaded, prestripped and surgeon-stripped DMEK grafts, respectively. Rebubbling rates were significantly associated with combined cataract surgery (p=0.009) and with time from harvesting the graft to the surgery (p=0.02).

**Conclusions:**

Decreased adhesion forces and elastic modulus in eye bank prepared tissues may contribute to increased rebubbling rates.

## INTRODUCTION

In the last decade, selective transplantation such as Descemet membrane endothelial keratoplasty (DMEK) that replaces damaged endothelium with a healthy donor endothelium has overtaken conventional full thickness transplantation due to clear advantages in terms of visual recovery, lower rates of rejection and faster rehabilitation.^1 2^ With this shift, new challenges have emerged such as consistency of tissue preparation and postoperative complications such as graft detachment.^3 4^ In order to overcome these issues and significantly reduce the learning curve^5^ (damage or wastage), eye banks have started to provide prestripped and/or preloaded tissue.^6–8^ In addition to less corneal wastage, this offers the advantages of tissue validation and better quality control. It has also been shown that endothelial graft preparation in the eye bank reduces surgeon effort and surgical time.^9^ This is particularly evident in the early stages of the learning curve.^5^ These advantages have led to a rapid increase in the popularity of eye bank prepared grafts.

Graft detachment, one of the most common but treatable complications after DMEK surgery, may affect the outcomes if not recognised early and properly managed. Although the management of graft detachment has improved,^3 10 11^ the causes are less clear. Apart from stripping, there is an additional step of preservation and transportation of the tissue that is required when an eye bank prepared DMEK, that is, prestripped or preloaded, is used, which may play a significant role in clinical outcomes. The purpose of this study, therefore, was to compare the adhesion forces, elastic modulus and rebubbling rates of prestripped, preloaded and surgeon-stripped DMEK grafts in order to identify factors associated with graft detachment.

## MATERIALS AND METHODS

### Ethical statement

The corneal tissues were procured by Fondazione Banca degli Occhi del Veneto (FBOV, Venice, Italy) with written consent from the donor’s next-of-kin to be used for transplantation and research purposes. Tissues used for research had lower endothelial cell counts (<2200 cells/mm^2^) but were otherwise healthy. All corneas used in the laboratory study were from FBOV and cultured/stored in the same media. The tissues were utilised and discarded as per the guidelines of Centro Nazionale Trapianti (Rome, Italy).

All the surgeries were performed at The Royal Liverpool University Hospital, Liverpool, United Kingdom by surgeons experienced in DMEK. The clinical study was approved by the Institutional Review Board (A0002786). All the tissues for transplantation were obtained from FBOV, Italy either as prestripped or preloaded DMEK.

### Laboratory investigation

#### Tissue evaluation

All the tissues (nine pairs of corneas) were randomly allocated to a group and stained with trypan blue (0.25% wt/vol; VisionBlue; D.O.R.C., Zuidland, The Netherlands) to evaluate the percentage of dead/necrotic cells before graft preparation. The endothelium was exposed to 1.8% hypotonic sucrose solution to aid counting of the endothelial cells and to examine its general morphology (pleomorphism and polymegathism). Endothelial cell density (ECD) was expressed as a mean of five different counts from each cornea using a 10×10 reticule mounted in the eyepiece of an inverted microscope (Axiovision, Zeiss, Oberkochen, Germany).

### Preparation of DMEK tissues

#### Prestripped DMEK

The tissues were stripped (n=6) following the method described previously by Parekh *et al.* ^8^ With the endothelium facing up, a 9.5 mm (Moria, Antony, France) punch was used to obtain a superficial trephination. After removing the excess peripheral membrane using 120 mm medium acute forceps (e.janach, Como, Italy), the tissues were stripped with a longitudinal movement using a 3-quadrant method, ensuring no torsions were generated during this phase to limit endothelial mortality.^12 13^ Once the tissues were stripped completely, they were replaced back on the corneal stroma and preserved in Cornea Jet (Eurobio, France) for in-house validation or shipped to Liverpool for further analysis.

#### Preloaded DMEK tri-folded with endothelium inwards

Following stripping as described above, the tissues (n=6) were tri-folded manually using acute forceps as described previously.^8^ The DMEK tissue was gently pulled into the preservation chamber of a 2.2 intraocular lens (IOL) cartridge (Viscoject, Wolfhalden, Switzerland) using a pair of Grieshaber Revolution 25 Gauge end-grasping forceps (Alcon, Ft Worth, Texas, USA) and preserved in Cornea Jet. The IOL cartridge was sealed with silicon plugs at both ends. Preloaded tissues were maintained in a sterile vial containing Cornea Jet and stored in-house for validation or shipped to Liverpool for further analysis.^6 8^ When required for analysis, silicon stoppers were removed, and the tissues were ejected from the funnel pore using the end-grasping forceps.

#### Surgeon-stripped DMEK

An IOL manipulator (Sinskey hook with blunt tip; Beaver-Visitec International Ltd., Warwickshire, UK) was used to score the peripheral circumference on the endothelial side of the tissues (n=6) detaching the periphery of the DMEK before 120 mm stripping straight pointed acute forceps (e.Janach) were used to strip the DMEK graft using a single peel (superior to inferior) method without using any dye. The cells on the tissue were kept moist with a single drop of phosphate-buffered saline (PBS) and were not totally submerged in the liquid. In challenging cases where the tissue was tightly attached to the stroma, the multiple quadrant method was used to avoid tissue tears.^13^ These tissues were not preserved and were analysed immediately.

#### Endothelial cell loss

After the preparation of tissues, the cells were stained with trypan blue for 20 s and placed in sucrose solution (1.8%) to visualise cell mortality and count the number of cells present after the preparation and preservation phase. Endothelial cell loss (ECL) was determined as a difference between the endothelial cell count before and after the preparation or preservation phase and after the subtraction of the trypan blue positive cells (TBPCs). This analysis was performed at FBOV for preloaded and prestripped tissues and in Liverpool for surgeon-stripped tissue.

#### Live/dead staining analysis

One graft from each donor was used for live/dead staining and the fellow graft for elastic modulus and adhesion force measurement. DMEK tissues (n=3 for each group) were triple stained to determine the viability of endothelial cells post transportation using Hoechst/ethidium homodimer/calcein AM (HEC) combination as previously described by Pipparelli *et al*.^14^ The DMEK tissues were first washed with PBS. Hoechst 33 342 (3 μg/mL, Thermo Fisher Scientific, Rochester, New York), ethidium homodimer EthD-1 (0.8 μM) and calcein AM (0.4 μM; LIVE/DEAD Viability/Cytotoxicity Kit, Thermo Fisher Scientific, Runcorn, UK) were added to PBS. Approximately 200 μL of the final solution was added to the completely stripped DMEK tissue on a glass slide and incubated at room temperature (RT) in the dark for 45 min. Relaxing radial cuts were made at three points to obtain a flat mount and tissue protected with a coverslip without mounting medium. HEC staining was viewed with an LSM 800 confocal microscope (Zeiss, Oberkocken, Germany). A tile scan was performed using a 5× objective and reconstructed using ZEN processing software to produce an image of staining across the whole surface of the graft. Trainable Weka Segmentation on Fiji was used to analyse the percentage area covered by viable cells, intermediate cells and denuded areas as previously described.^15 16^

#### Elastic modulus and adhesion force measurement

The DMEK tissues (n=3 for each group) were washed with PBS and fixed on circular glass coverslips (12 mm diameter), which were attached to metal disks for mounting into the atomic force microscope (AFM). Elastic modulus and adhesion force of the anterior surface of the tissues were measured using a Bruker MultiMode 8 AFM (Bruker Nano Inc., Nano Surfaces Division, CA). The AFM was uploaded with a silicon probe with a rectangular tip, type RTESPA-300 (Bruker Nano Inc., CA). The PeakForce quantitative nanomechanical mapping (PF-QNM) mode in air with the Derjaguin-Muller-Toporov (DMT) model were utilised as previously described in the literature.^17 18^ The relative calibration method for PF-QNM was performed before every test. A clean sapphire sample (Sapphire-12 M; Bruker Nano Inc., Nano Surfaces Division, CA) and a Vishay Photostress PS1 Polymer reference sample (Vishay; Wendell, NC) were utilised in the calibration process. The PS1 had a known elastic modulus of 2.7±0.1 GPa. During calibration, adhesion force was maintained at less than 1 nN on the sapphire sample. The tip radius was maintained at 20 nm in all experiments.

AFM images of the DMEK tissues were captured from six different locations (three at the centre and three at the mid-periphery) on each sample. The centre of the samples was visually identified using the optical microscopy integrated with the AFM and scanned in three places approximately 500 µm of each other. Another three places were scanned at the mid-periphery of the samples, 3.5 mm from the first central scans. Image scanning size was set to 1 µm, where 256 horizontal lines in each image were captured. All images were scanned at a scan rate of 0.666 Hz, and a resolution of 256 pixel/line. The peak force frequency and amplitude were set to 2 kHz and 150 nm. Elastic modulus and adhesion force were measured from the AFM images of the DMEK tissues after processing the images using NanoScope Analysis 1.8 software (Bruker Nano Inc., Nano Surfaces Division, CA).

#### Clinical investigation

In this retrospective case series, all records from patients treated for endothelial dysfunction (Fuchs endothelial corneal dystrophy (FECD) or pseudophakic bullous keratopathy (PBK)) with a DMEK between March 2017 and October 2019 were analysed. Exclusion criteria were patients who had glaucoma or had glaucoma surgery, previous corneal transplants, abnormal anterior segment, previous uveitis and patients without anterior segment optical coherence tomography (OCT) in the first postoperative week. Twenty-seven eyes were excluded as per this exclusion criteria. Surgery was performed by three surgeons all who had significant experience in lamellar surgery (at least 30 surgeons prepared DMEK procedures and at least 20 preloaded). The surgeon-stripped DMEKs were prepared at the same time as the surgery in the same operating room. The prestripped or preloaded tissues were prepared and shipped from FBOV to The Royal Liverpool University Hospital. In the combined cataract procedure, the lens replacement was always performed before the graft was introduced. Following DMEK delivery, all the tissues were attached with air tamponade. In case of graft detachment, the patients were rebubbled if the detachment was more than 30% or involved the visual axis. Data such as gender, age at the time of the surgery, primary diagnosis, donor details, time from harvesting to surgery, surgery details (graft diameter and combination with phacoemulsification), best corrected visual acuity (BCVA) and postoperative complications (such as air release and rebubbling rate) were recorded.

### Statistical analysis

Data are presented as mean ± SD or median (IQR; IQR) for continuous variables, or as percentages for categorical variables. A two-tailed non-parametric Wilcoxon signed-rank test for elasticity and adhesion of the same group was carried out with 95% CI. Non-parametric Kruskal-Wallis test with Dunn’s post hoc test with significance level of alpha =0.05 (95% CIs) was used to compare the data between all the groups using Prism 5 software (GraphPad, San Diego, CA).

Preoperative and postoperative BCVA were tested for normality using the Shapiro-Wilk test. Multi-group comparisons were performed with ANOVA or Kruskal-Wallis, if they were quantitative or categorical data, respectively, followed by Tukey’s and Dunn’s post hoc analysis. For the analysis of the different rebubbling rates among the groups, considering the small number of prestripped DMEK, a Fisher’s exact test was used. A generalised linear model was fit with predictors through backward elimination controlling for confounders to identify risk factors associated. The statistical analyses were performed using STATA 14.0 (StataCorp, College Station, TX) and a p value <0.05 was considered statistically significant for all the tests.

## RESULTS

### Laboratory investigation

#### Donor characteristics

Mean age of the donors was 69.8±7.9 years (6 males and 3 females) with postmortem time of 15.8±8.7 hours. The tissues were stored in tissue culture media in the eye bank for 27±8.9 days before use. The time between preparation of eye bank prepared tissues and analysis in Liverpool was 3 days. None of the donors were diabetic.^19^

#### Endothelial cell loss

**Figure 1:**
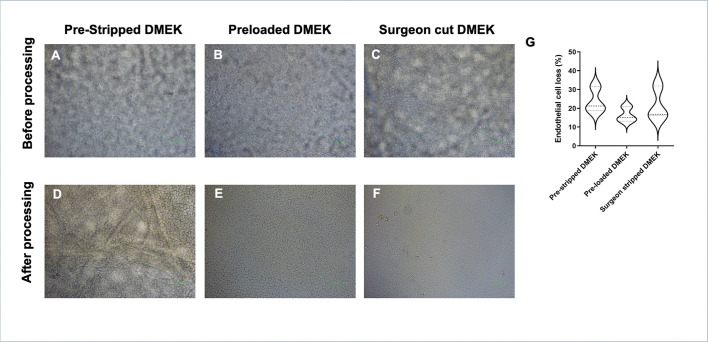
Corneal endothelial cell density and morphology determined using trypan blue staining comparing the tissues before processing for (A) prestripped DMEK, (B) preloaded DMEK endo-in and (C) surgeon-stripped DMEK grafts and after processing for (D) prestripped DMEK, (E) preloaded DMEK endo-in and (F) surgeon-stripped DMEK grafts. (G) Endothelial cell loss comparing all the groups. The data are represented as in a violin plot showing median (dashed line) and quartiles (dotted lines) (Kruskal-Wallis test).

The endothelial cell morphology was good and trypan blue positivity low (n=18) both before and after processing the tissue for prestripped DMEK (figure 1A, D), preloaded DMEK (figure 1B, E) and surgeon-stripped DMEK (figure 1C, F). ECL of 23.8±6.7% was observed in the prestripped DMEK group compared with 16.3±4.1% in the preloaded DMEK group and 21.6±8.9% in the surgeon-stripped DMEK group, which was not a statistically significant difference (p=0.25) (figure 1G).

#### Live/dead staining

**Figure 2:**
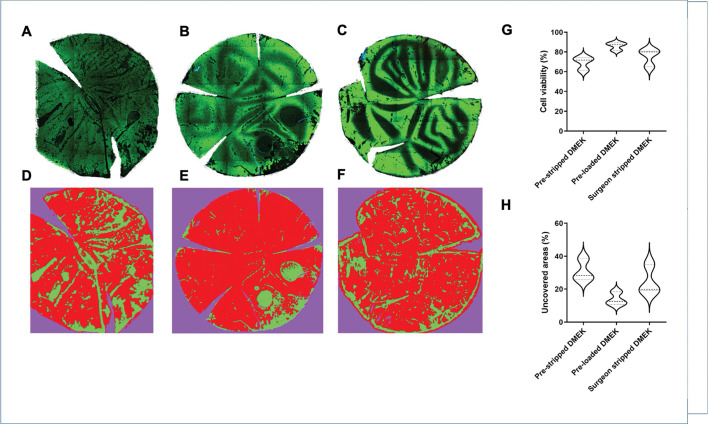
Live/dead analysis using HEC staining (A–C) and Weka segmentation (D–F) on (A, D) prestripped DMEK, (B, E) preloaded DMEK endo-in and (C, F) surgeon-stripped DMEK grafts. (G) Overall cell viability and (H) uncovered areas between all the groups. The data are represented in violin plots showing median (dashed line) and quartiles (dotted lines) (Kruskal-Wallis test).

The majority of cells in prestripped DMEK (n=3; figure 2A, D), preloaded DMEK (n=3; figure 2B, E) and surgeon-stripped DMEK (n=3; figure 2C, F) were calcein AM positive viable cells with minimal ethidium homodimer positive dead cells seen in all grafts. Uncovered areas were seen mostly at the periphery and in fold lines observed as an absence of all staining, including nuclear Hoechst staining. The percentage of viable cells was 69.1±6.7% in the prestripped DMEK group compared with 86.1±4.1% in the preloaded DMEK and 75.3±9.0% in the surgeon-stripped DMEK group, which was not a statistically significant difference (p=0.051; figure 2G). There was no statistically significant difference in the uncovered area (area without cells) in the prestripped DMEK group, which was 30.86±6.8%, compared with 13.89±4.1% in the preloaded DMEK and 24.68±8.9% from the surgeon-stripped DMEK group (p=0.051; figure 2H).

#### Elastic modulus

**Figure 3:**
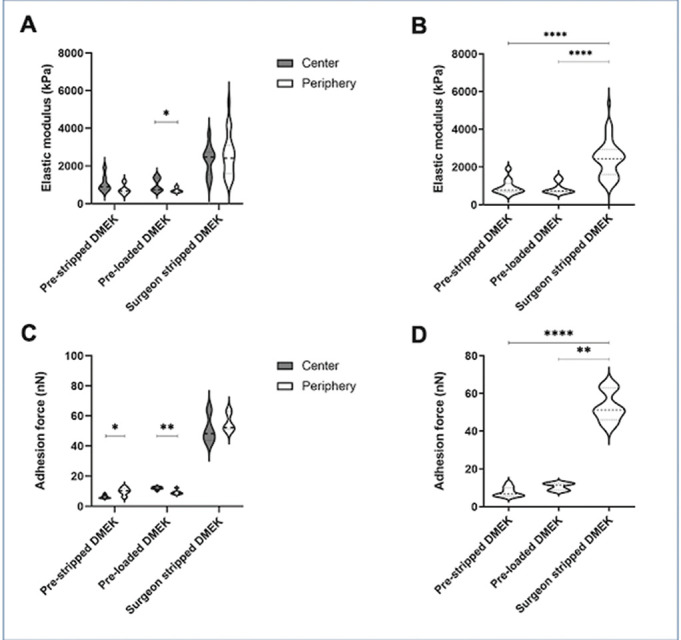
Elastic modulus in (A) the centre and mid-periphery of DMEK grafts. (B) Comparison of elastic modulus in the entire tissue between all the groups. (C) Adhesion force in the centre and mid-periphery of DMEK grafts. (D) Comparison of adhesion force in the entire tissue between all the groups. The data are represented in violin plots showing median (dashed line) and quartiles (dotted lines) (A, C) Wilcoxon test and (B, D) Kruskal-Wallis test. *p<0.05, **p<0.01, ****p<0.0001.

The elastic modulus (n=9; n=3 from each group) was significantly higher in surgeon-stripped DMEK compared to the prestripped DMEK (p=0.0001) or preloaded DMEK groups (p=0.0001; figure 3A, B). The elastic modulus, however, was similar in the centre to the mid-periphery for the prestripped DMEK (1059±433 MPa and 748±258 MPa, respectively, p=0.073) and surgeon-stripped DMEK groups (2305±777 MPa and 2577±1114 MPa, respectively, p=0.54). The elastic modulus in the centre of the preloaded DMEK (1014±347 MPa), however, was significantly higher than in the mid-periphery 714±119 MPa (p=0.015).

#### Adhesion force

The adhesion force is the force required to lift the tip of the cantilever from the surface of the graft. Mean adhesion force (n=9; n=3 for each group) was significantly lower in the centre compared to the mid-periphery for the prestripped tissue (6.3±1.0 nN vs 9.9±2.4 nN, p=0.023) but significantly higher in the centre for the preloaded tissue (12.1±0.8 nN vs 9.2±1.4 nN, p=0.002; figure 3C). There was, however, no difference in the mean adhesion force between the centre (50.8±9.3 nN) and mid-periphery (55.5±6.5nN) in the surgeon-stripped DMEK group (p=0.40; figure 3C). The mean adhesion force in the centre of surgeon-stripped DMEKs was significantly higher compared with prestripped DMEK or preloaded DMEK (p=0.0003); however, in the mid-periphery it was significantly higher only between the surgeon-stripped DMEK group and preloaded DMEK group (p=0.0225). Overall, surgeon-stripped DMEK grafts showed significantly higher adhesion force compared with prestripped (p<0.0001) and preloaded groups (p<0.0084; figure 3D).

#### Clinical investigation

Ninety-one eyes (39 male and 52 female) of 79 patients with a mean age of 74.3±9.2 years were analysed. Sixty-three eyes presented with FECD and 27 eyes with PBK. Forty eyes underwent surgeon-stripped DMEK, 20 eyes had prestripped DMEK and 31 eyes received preloaded DMEK. Mean donor age was 67.6±8.3 years and mean time from graft harvesting to surgery was 0.16±0.4 days for surgeon-stripped DMEK, 3.34±0.8 days for prestripped tissues and 3.13±0.8 days for preloaded tissue. Grafts had an average diameter of 8.65±0.5 mm (8.0–9.5 mm) and a preoperative ECD of 2545.5±113.6 cells/mm^2^.

Fifty-nine per cent of patients (54 eyes) had phacoemulsification of the lens and intraocular lens implant combined with DMEK surgery (table 1). Preoperative BCVA (0.72±0.57 LogMAR) improved significantly after the surgery (0.36±0.47 LogMAR, p<0.01). Average time to reach BCVA was 101.4±115.3 days; 54.5% of patients had some form of post-op graft detachment and in 32% of cases it required a rebubbling procedure. The rebubbling rate of prestripped DMEK was 40%, preloaded DMEK 48.4% and surgeon-stripped DMEK 15.4%. Kruskal-Wallis analysis highlighted a statistical difference between the groups (p=0.009) and post hoc analysis revealed a significant difference between surgeon-stripped DMEK and prestripped DMEK (p=0.03), and between surgeon-stripped DMEK and preloaded DMEK (p=0.002). A subanalysis of the re-bubbling rate with and without combined cataract surgery revealed a significant difference between surgeon-stripped DMEK and preloaded DMEK (p=0.02 combined and p=0.01 in DMEK surgery only), and a difference between surgeon-stripped DMEK and prestripped DMEK in the DMEK only surgery (p=0.04). There was no difference in BCVA improvement between the three groups (table 1). Rebubbling rate was significantly associated with combined cataract surgery (p=0.006) and time from harvesting to surgery (p=0.03). We found no association between BCVA and any of the other factors analysed: re-bubbling (p=0.44), time from graft harvesting to surgery (p=0.74) and combined cataract surgery (p=0.29). There were, however, fewer patients with PBK compared to FED, in surgeon prepared (FED 21, PBK 19), preloaded and prestripped (FED 42 PBK 9) groups and this may account for insufficient power to detect a potential difference in rebubbling rates or postoperative visual acuity between PBK and FED.

**Table 1:** Summary of patients and outcome analysis for all three groups

	ps-DMEK	pl-DMEK	ss-DMEK	P value
**Patient details**				
N	20	31	40	
Gender (M/F)	10/10	10/21	19/21	0.3
Age (years)	73.8±9.5	77.1±9.2	72.4±8.6	0.09
**Outcome analysis**				
Rebubbling	40%	48.40%	15.40%	**0.009**
Combined cataract surgery	80%	61.30%	47.50%	0.06
Preoperative BCVA (LogMAR)	0.59±0.46	0.86±0.65	0.68±0.56	0.23
Postoperative BCVA (LogMAR)	0.29±0.34	0.41±0.52	0.35±0.48	0.65

Bold text shows a statistically significant difference among the groups.

ps-DMEK, prestripped DMEK; pl-DMEK, preloaded DMEK; ss-DMEK, surgeon-stripped DMEK.

The postoperative follow-up time was 12.4±9.1 (range 3–20.5) months. No eyes had developed immunological rejection at last follow-up and all the patients were receiving topical steroids. Two patients underwent subsequent regrafting (DMEK) due to failure.

## DISCUSSION

Preloaded and prestripped DMEK tissues provide several benefits to the surgeon including standardised preparation, time saving and assurance that each patient will receive validated tissue.^6^ Graft detachment and rebubbling rates, however, remain a concern after delivery of preloaded DMEK.^3^ In our multi-surgeon setting study, we report that the overall incidence of any degree of graft detachment was 54.5% with 32% requiring at least one re-bubbling when more than one-third of the graft was detached. The rebubbling rate was significantly higher in the preloaded and prestripped DMEK groups versus the surgeon-stripped DMEK group.

We have shown using an AFM that the adhesion force is greater in the surgeon-stripped group when compared to prestripped and preloaded, which may explain why the rebubbling rate was much lower in the surgeon-stripped group than the other two groups. Most DMEK detachment is initiated from the periphery of the grafts so we did expect that the adhesion force at the periphery would be less compared to the centre; however, we found no consistent difference in our study. This may have been because we were limited by our testing equipment to only testing the mid-periphery (3.5 mm from the centre) rather than the extreme periphery.

It is also worth highlighting that the elastic modulus (stiffness) was significantly lower in preloaded and prestripped tissues and this may also have an influence on DMEK graft attachment. The reason for the difference in stiffness and adhesion force is unknown but we hypothesise that the longer exposure time of the DMEK tissues to dextran containing media may be associated with this higher rate of detachment. Surgeon-stripped DMEK grafts are prepared and transplanted without undergoing any additional preservation phases. This allows the tissue to stay in its natural conformation, attached to the stroma, without exposing the Descemet’s membrane (DM) directly to the constituents of the shipping medium. Conversely, prestripped and preloaded DMEK tissues are preserved in dextran-based media after preparation with their DM directly exposed to the fluid. Dextran is a complex branched glucan (a polysaccharide derived from the condensation of glucose) and is used as a hypertonic solution to restore the correct corneal thickness by removing excess fluid from the tissue. It is possible that direct exposure of the DM to the dextran, especially in the preloaded tissue which is folded with endothelium in, may result in the deposition of a thin film on the DM that may interfere with the adhesion of the graft to the stroma resulting in an increased likelihood of graft detachment. Schlötzer-Schrehardt *et al* described the interfacial matrix that is present at the cleavage plane between the stroma and the DM when the DM-endothelial cell layer is stripped from a donor.^20^ This interfacial matrix varies between donors and is comprised of connecting collagen fibres and proteoglycan-like filaments. We suggest it is the disruption of this interfacial matrix after prolonged exposure to dextran containing medium in the preprepared tissues that leads to an increased rate of detachment in those groups.

We believe that the tri-folding of tissues in the endothelium-in configuration in this study exposes the DM to dextran to a greater extent when compared with prestripped or surgeon-stripped tissues. Newman *et al* reported a case series of preloaded DMEK (endothelium-out) stored in the Straiko modified Jones tube at the eye bank and delivered in an Optisol-filled viewing chamber to the surgeon for transplantation 1 to 2 days later. They reported a rebubbling rate of only 14.4.% compared to our 48% for preloaded tissues.^7^ The differences between the studies were a lower concentration of dextran (1% compared to 6% in our study), conformation of the preloaded DMEK (endothelium out) and the time from preparation to delivery. Although the grafts were washed before insertion or testing, perhaps more thorough washing of the prestripped tissue to remove any remnant of dextran containing medium may improve the attachment rate. The endothelium-in conformation does offer advantages such as easier and more predictable graft unfolding but washing would not be possible as it is preloaded; therefore, reduction in the amount of dextran in the transport medium or its complete elimination may reduce the detachment rate with these tissues.

In our study, cataract surgery was a risk factor for detachment of the corneal graft; however, this was independent of whether the graft was surgeon prepared or eye bank prepared. Conversely, in a larger study by Chaurasia *et al*, it was demonstrated that there was no additional risk of significant detachment requiring rebubbling with cataract surgery. In that study, rebubbling was only performed if the detachment was worsening or affecting the patient’s vision. Over 292 patients who had DMEK alone and 200 that underwent DMEK triple procedures, all of them being surgeon prepared, they showed a similar rate of graft detachment needing rebubbling (30% vs 29%) (p=0.69).^21^ Leon *et al* ^22^ demonstrated high detachment and rebubbling rates of 86.4% for DMEK triple and 50.9% for DMEK alone. These results, in addition to our data, raise potential concerns regarding preloaded DMEK, which we have identified as having the highest rebubbling rate.

This study is limited by its retrospective design and we also recognise that an endothelial cell count in the postoperative follow-up would have been useful, but those data were only available for a small number of the cohort and therefore were not analysed. Although the number of procedures performed by each surgeon was not the same for each type of graft, because all surgeons were experienced in both techniques we do not believe that this would have significantly influenced the outcomes. In our laboratory study we observed that although the preparation conditions may have affected the adhesion force and elastic modulus, they did not influence ECL and cell viability. Specifically, the preloaded DMEK group showed minimal ECL, the highest cell viability and smallest denuded area compared to the other groups. These results are in line with our previous findings, where tri-folded, preloaded DMEK following shipping, showed similar levels of ECL.^16^

In conclusion, our data show a higher detachment rate and rebubbling rate in preloaded (with endothelium-in) and prestripped DMEK. The rebubbling rate does not affect the visual acuity achieved and the speed of recovery. We suggest that, for tissues prepared by eye banks, the time from graft preparation to surgery should be kept to a minimum because this may influence the rebubbling rate. Although surgeon-stripped preparation may reduce the detachment rate compared to the eye bank prepared tissues, the advantages such as less tissue wastage, reduced surgical time and theatre costs must be considered when choosing which type of graft to use for transplantation purposes.
